# Improving the monitoring of root zone soil salinity under vegetation cover conditions by combining canopy spectral information and crop growth parameters

**DOI:** 10.3389/fpls.2023.1171594

**Published:** 2023-07-04

**Authors:** Xiaoyan Shi, Jianghui Song, Haijiang Wang, Xin Lv, Tian Tian, Jingang Wang, Weidi Li, Mingtao Zhong, Menghao Jiang

**Affiliations:** ^1^ College of Agriculture, Shihezi University, Shihezi, China; ^2^ Key Laboratory of Oasis Ecological Agriculture of Xinjiang Production and Construction Corps, Shihezi University, Shihezi, Xinjiang, China

**Keywords:** canopy hyperspectral data, growth parameters, partial least squares regression, soil salinization, variable selection

## Abstract

Soil salinization is one of the main causes of land degradation in arid and semi-arid areas. Timely and accurate monitoring of soil salinity in different areas is a prerequisite for amelioration. Hyperspectral technology has been widely used in soil salinity monitoring due to its high efficiency and rapidity. However, vegetation cover is an inevitable interference in the direct acquisition of soil spectra during crop growth period, which greatly limits the monitoring of soil salinity by remote sensing. Due to high soil salinity could lead to difficulty in plants’ water absorption, and inhibit plant dry matter accumulation, a method for monitoring root zone soil salinity by combining vegetation canopy spectral information and crop aboveground growth parameters was proposed in this study. The canopy spectral information was acquired by a spectroradiometer, and then variable importance in projection (VIP), competitive adaptive reweighted sampling (CARS), and random frog algorithm (RFA) were used to extract the salinity spectral features in cotton canopy spectrum. The extracted features were then used to estimate root zone soil salinity in cotton field by combining with cotton plant height, aboveground biomass, and shoot water content. The results showed that there was a negative correlation between plant height/aboveground biomass/shoot water content and soil salinity in 0-20, 0-40, and 0-60 cm soil layers at different growth stages of cotton. Spectral feature selection by the three methods all improved the prediction accuracy of soil salinity, especially CARS. The prediction accuracy based on the combination of spectral features and cotton growth parameters was significantly higher than that based on only spectral features, with R^2^ increasing by 10.01%, 18.35%, and 29.90% for the 0-20, 0-40, and 0-60 cm soil layer, respectively. The model constructed based on the first derivative spectral preprocessing, spectral feature selection by CARS, cotton plant height, and shoot water content had the highest accuracy for each soil layer, with R^2^ of 0.715,0.769, and 0.742 for the 0-20, 0-40, 0-60 cm soil layer, respectively. Therefore, the method by combining cotton canopy hyperspectral data and plant growth parameters could significantly improve the prediction accuracy of root zone soil salinity under vegetation cover conditions. This is of great significance for the amelioration of saline soil in salinized farmlands arid areas.

## 1 Introduction

In recent decades, global warming and anthropogenic activities have continuously caused the Earth system to deviate from its normal state. In particular, soil degradation accelerates, leading to a decline in soil quality. Soil salinization is one of the main causes of land degradation in arid and semi-arid areas. It not only restricts the sustainable agricultural development, but also greatly threatens regional ecological environment. Therefore, soil salinization has attracted many attentions from scholars. In Xinjiang, China, the special topography and arid climate have led to increasing soil salinization (Zhang et al., 2021a), which greatly limits the development of agriculture and animal husbandry. For example, in the Manas River Basin in Xinjiang, the flat terrain, high parent material salinity, arid climate, and strong evaporation have caused the massive accumulation of salts in the agricultural fields. In addition, the long-term irrigation results in the rise of groundwater level. Therefore, soil salinization and secondary salinization are very prominent. To ameliorate saline soils, accurate monitoring of soil salinity changes is very necessary.

Conventional soil salinity determination mostly relies on in-situ sampling and indoor experiment. These methods are not only labour-intensive and time-consuming, but also cannot accurately and timely reflect the changes of soil salinity in vast lands. Hyperspectral remote sensing, with the fast, nondestructive, and high efficiency characteristics, has become a hotspot in the study of soil salinization monitoring. Spectral features of soil salinity can be directly obtained by remote sensing under bare soil conditions, and the spectral reflectance increased with the increase of soil salinity at visible-near infrared region (Sidike et al., 2014; El Harti et al., 2016). However, soil spectra acquisition is highly influenced in areas covered with vegetation, which inhibits direct spectral monitoring of soil salinity. To solve this problem, academics have done a great deal of researches. Several scholars removed areas with high vegetation coverage, which led to information loss (Wester et al., 1990). Some studies also used vegetation index to invert soil salinity (Ramos et al., 2020; Wang et al., 2020; Zhang et al., 2021a; Zhu et al., 2021). However, specific vegetation indices usually use reflectance of limited bands for estimation, ignoring the hyperspectral information in other bands. Besides, the characterization in vegetation indices will be delayed when crops are subjected to salt stress, which may lead to problems such as low estimation accuracy and poor universality (Liu et al., 2019). Hyperspectral remote sensing data provide complete information of crop spectrum, which makes it possible to monitor soil salinity. However, the increase of uncorrelated bands will also bring about the increase of information redundancy and complexity. Therefore, it is necessary to eliminate the noise and redundancy of canopy hyperspectral data and select appropriate spectral features in the monitoring of soil salinity under vegetation cover conditions.

In spectral prediction, the inevitable inclusion of interference and irrelevant information such as noise in the spectral data, and the collinearity between variables will affect the robustness and prediction accuracy. Variable selection methods such as Competitive Adaptive Reweighted Sampling (CARS), Genetic Algorithm (GA), Random frog algorithm (RFA), Successive Projections Algorithm (SPA), Uninformative Variable Elimination (UVE), and Variable Importance in Projection (VIP), could extract spectral features from massive and complex data, so as to simplify multivariate models and improve prediction accuracy and robustness (Sun et al., 2021a; Kamruzzaman et al., 2022). For example, Yang et al. (2012) used spectral techniques to quantitatively analyze soil nitrogen and carbon at farm scale and found that the UVE method could effectively eliminate invalid information and increase prediction accuracy. Vohland et al. (2014) used the CARS method to extract spectral features to establish a PLSR (Partial Least Squares Regression) model for soil organic matter content estimation, and found that the PLSR model based on CARS had a higher prediction accuracy than the PLSR model constructed using the full band. Peng et al. (2014) accurately predicted soil organic carbon content by combining SPA and support vector machine regression (SPA-SVMR), with R^2^ of 0.73. However, different spectral feature selection methods have different prediction accuracy, that is, the selected spectral features and their numbers are different (Zou et al., 2010; Vohland et al., 2014). Besides, different feature selection methods have different applicability for different data sets. Therefore, it is necessary to determine the optimal feature selection method for a data set in spectral prediction.

Cotton (*Gossypium hirsutum* L.) is the most important cash crop in Xinjiang, China, and the widespread application of drip irrigation and plastic film mulching make cotton roots mainly distributed in the 0-60 cm soil layer (Min et al., 2014). Previous studies have shown that the salinity in root zone directly affects cotton growth parameters such as plant height, aboveground biomass, and moisture content (Abdelraheem et al., 2015a; Abdelraheem et al., 2015b; Du et al., 2016; Oluoch et al., 2016; Abdelraheem et al., 2019). For example, Ahmad et al. (2002) found that high soil salinity inhibited the growth of cotton roots and shoots, increased bud abscission, and decreased the boll number per plant. At present, the saline soil amelioration methods mainly include the development of salt-tolerant crop varieties (Kumar et al., 2017), the application of amendments (Kibria et al., 2015), and engineering measures (Shi et al., 2021). In arid areas in Xinjiang, the high groundwater level and poor water quality are the main reasons for the accumulation of salts in the tillage layer of farmlands, and reducing the groundwater level and the upward movement of salts in groundwater is very necessary to inhibit soil salinization. Therefore, salt isolation layer in root zone and subsurface drainage have been applied to inhibit soil salinization in farmlands. Studies have shown that salt isolation layer in root zone can effectively inhibit the upward movement of salts with groundwater by blocking soil capillaries (Starr et al., 1978), and the subsurface drainage can effectively reduce the groundwater level and the risk of secondary salinization through drainage (Ritzema et al., 2006).

At present, the effects of saline soil amelioration measures can be directly evaluated by measuring soil salinity and indirectly evaluated by monitoring the growth parameters of crops. Directly measuring soil salinity in cotton fields during cotton growth season is time-consuming and laborious, while remote sensing provides a good option for monitoring. However, how to use remote sensing technology to accurately obtain crop root zone soil salinity information under vegetation cover conditions is an urgent problem to be solved. At present, most studies on spectral monitoring of soil salinity only consider the spectral features of soil salinity, vegetation index, etc. (Wu et al., 2021; Zhu et al., 2021; Chen et al., 2022; Jia et al., 2022), without considering the combination of crop growth parameters and spectral information. Therefore, this study hypothesized that the combination of crop canopy spectral information and crop growth parameters (cotton plant height, aboveground biomass, and shoot water content) might improve the estimation accuracy of soil salinity in root zone under vegetation cover conditions. The objectives were to: (1) study the effects of soil salinity on cotton growth parameters at different growth stages; (2) compare the effects of different feature selection methods (VIP, CARS, and RFA) on the spectral prediction accuracy of salinity of different soil layers (0-20 cm, 0-40 cm, and 0-60 cm); and (3) explore the potential of combination of canopy spectral data and crop growth parameters in the monitoring of root zone soil salinity, to obtain a universal soil salinity monitoring method.

## 2 Materials and methods

### 2.1 Study site

**Figure 1:**
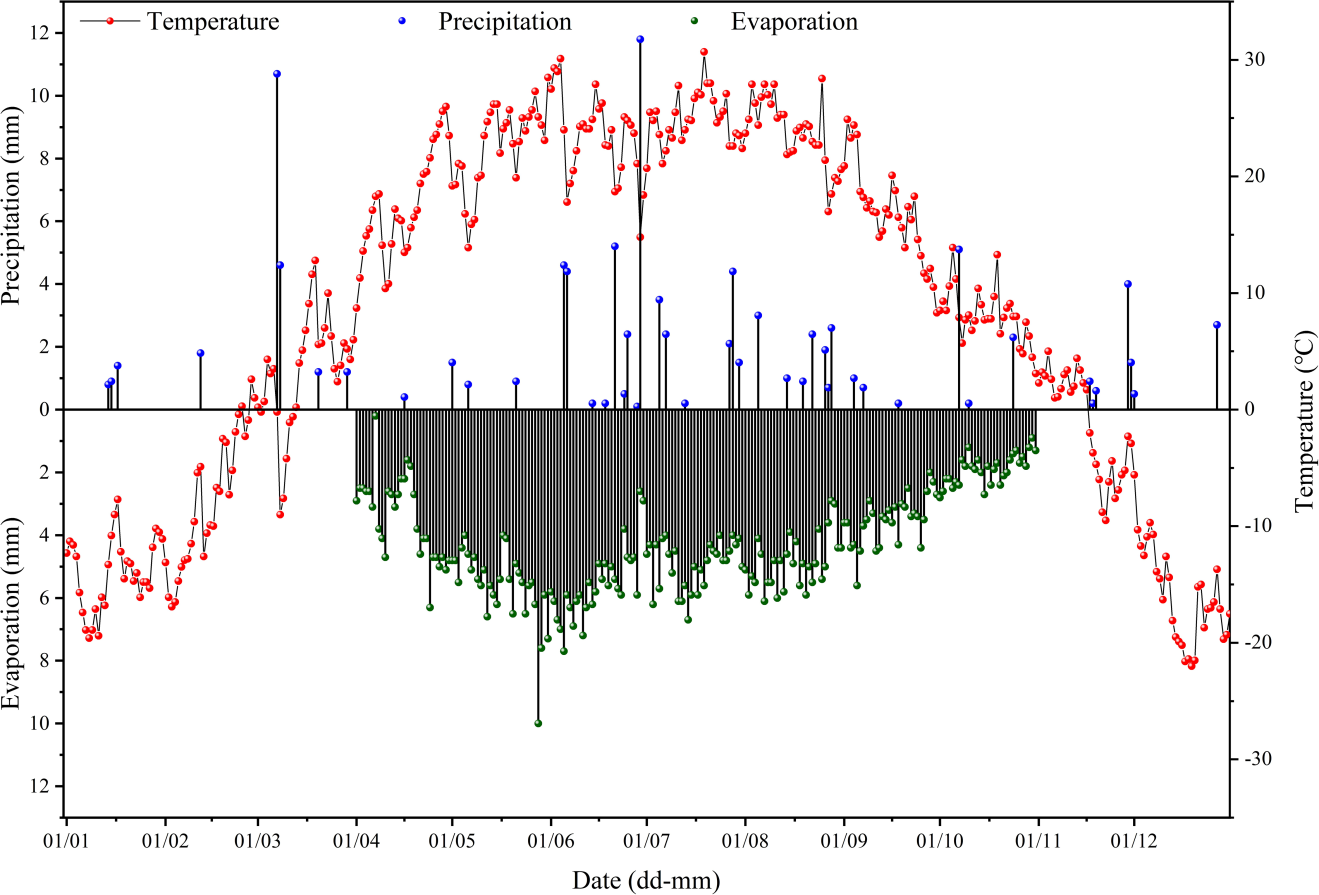
Variation of temperature, precipitation, and evaporation in the study area in 2020.

The research area is located in an experimental base in Beiwucha Town, Manas County, Xinjiang, China (44°35′N, 86°15′E). The site has a temperate continental climate with hot summer and cold winter. The average annual temperature was 6.8°C and average annual precipitation was 152.6 mm. The average annual evaporation (1967 mm) was much greater than average annual precipitation (**Figure 1**). The terrain was flat and the soil type was meadow soils. The soil texture was primarily medium loam, with a large thickness. The groundwater level was high, and the average annual groundwater level was between 0.5 and 3.0 m. The mineral concentration of groundwater was greater than 30 g L^-1^. The soil salinity of the study site was very high (6.84 dS m^-1^ (EC_1:5_) in the 0-20 cm soil layer) before construction of the base in 2011. And salinity of groundwater in the study area was greater than 30 g L^-1^. The irrigation water was from a nearby reservoir, with a salinity below 1 g L^-1^ (Wang et al., 2021).

### 2.2 Test design

In 2011, three saline soil amelioration treatments including salt isolation by a stone layer combined with cropping (T1), subsurface drainage combined with cropping (T2), cropping (T3) and a control group (CK) were set up (**Table 1**). Cotton was cropped since 2012 except for 2014 and 2015 (the field was abandoned for lack of irrigation water). In 2018, cotton was cropped in part of the site of the CK, which was recorded as T4 treatment. There were significant differences in soil salinity among treatments before sowing in 2020 (**Table 1**), which provided a good experimental condition for this study. In 2011, the initial average soil salinity (EC_1:5_) of the 0-20, 20-40, and 40-60 cm soil layers were 6.84, 6.96, and 6.83 dS m^-1^, respectively, indicating severe soil salinization (**Table 2**). After 9 years of saline soil remediation, there were differences in soil salinity among different soil layers before cotton sowing in 2020, providing a good experimental condition for this study. The detailed irrigation timing, irrigation quota, and nitrogen fertilizer application amount are shown in **Table 3**.

**Table 1:** Details for treatments in the experiment.

Treatment	Details
T1	A salt isolation stone layer was built to stop the upward movement of salt in the deep soil layer by blocking the soil capillary. In 2011, the 0-20, 20-40, 40-60, 60-80, 80-100, and 100-120 cm soil layer of a plot (100 m × 70 m) were excavated in turn (the bottom slope was 1: 500), while the bulk density of each soil layer was measured. Then, the pit bottom was covered with a geotextile, and the sides were covered with a waterproof plastic cloth. After that, stones (particle size: 2-4 cm) were covered on the geotextile (thickness: 15-20 cm). A geotextile was then covered on the stone layer. Finally, the soils were backfilled. To ensure consistent bulk density, the soils of different layers were compacted until the bulk density reached the initial value. The details for cropping are shown in T3 treatment.
T2	Subsurface drainage was designed according to the principle of “salt moves with water”. In 2011, corrugated drainage pipes with inner diameter of 110 mm were wrapped with geotextile after being punched, and laid by a trenching machine in a plot (100 m × 70 m). The space and layout depth of drainage pipe were 10 cm and 100 cm, respectively, and the slope was 1:500. The drainage pipes were connected with the water collecting pipe through a gate valve. The water in the water collecting pipe was discharged to the drainage channel outside the study area through the built water collecting tank. The details for cropping are shown in T3 treatment.
T3	Cotton (cv. Xinluzao 79) was sown in April, and drip irrigation and plastic film mulching were adopted. Three drip pipes and six rows were under each film. Full irrigation was applied 11 times during the whole growth period, with the quota of 480 mm. Besides, 105 kg ha^-1^ of P_2_O_5_, 60 kg ha^-1^ of K_2_O, and 72 kg ha^-1^ of pure N were basally applied, and the remaining 288 kg ha^-1^ of pure N was topdressed with irrigation water in different periods. Irrigation was stopped on 30^th^ Aug (135 days after sowing), and irrigation was conducted 1, 3, and 7 times at the seedling, bud, and flowering and boll-forming stage, respectively. Other field managements were consistent with local practice. The area of the plot was 100 m × 70 m.
T4	In 2018, cotton was cropped in part of the site of the CK, which was recorded as T4 treatment (80 m × 70 m). The details for cropping are shown in T3 treatment.
CK	Bare land (Before 2017, the area of the plot was 165 m × 70 m; After 2018, it was 85 m × 70 m)

**Table 2:** Details for treatments in the experiment.

Treatments	Initial soil salinity in 2011 (dS m^-1^)				Soil salinity in 2020 (dS m^-1^)			
	0-20 cm	20-40 cm	40-60 cm	60-100 cm	0-20 cm	20-40 cm	40-60 cm	60-100 cm
T1	6.80a	6.93a	6.85a	6.81a	2.89ab	3.23ab	3.10ab	3.04ab
T2	6.82a	6.95a	6.85a	6.76a	1.97b	2.62b	2.67b	2.41b
T3	6.88a	6.97a	6.81a	6.77a	2.45ab	2.72b	3.37ab	3.25ab
T4	6.85a	6.99a	6.82a	6.74a	3.47a	3.74a	3.78a	3.65a

Different lowercase letters in each column indicate significant difference (at the 95% confidence interval) between treatments in the same soil layer.

**Table 3:** Information of irrigation and N topdressing.

Growth stage	Irrigation timing (days after sowing)	Irrigation quota/mm	Topdressed N/%
Seedling stage	45	30	3
Squaring stage	61	40	6
	71	40	10
	79	40	15
Flowering and bolling stage	86	50	15
	92	50	15
	97	50	12
	102	50	12
	108	50	6
	122	40	4
	135	40	2

### 2.3 Data collection

Cotton canopy spectral reflectance were collected in-situ using a high spectral resolution portable spectroradiometer SR-3500 (Spectral Evolution Inc., Lawrence, MA, USA) from 11:00 to 15:00 on 6^th^ July (full-budding stage; 76 days after sowing (DAS)), 6^th^ Aug (full-flowering stage; 111 DAS), 19^th^ Aug (boll-setting stage; 124 DAS), 6^th^ Sep (initial boll-opening stage; 142 DAS), and 26^th^ Sep (boll-opening stage; 162 DAS). The instrument has a spectral range of 350-2500 nm, and the spectral resolution for 350 ~ 1000 nm, 1000 ~ 1500 nm, and 1500 ~ 2500 nm were 3.5 nm, 10 nm, and 7 nm, respectively. The spectral sampling interval was 1 nm. During spectral acquisition, a non-contact fiber optic probe was placed 100 cm above the cotton canopy, with a field of view of 25°. A white barium sulfate (BaSO_4_) panel was used for calibration before each spectral acquisition. 7 replicates were selected from each treatment for spectral acquisition, and 5 canopy data were collected for each replicate. After removing the abnormal data, the mean of each point was used as the spectral reflectance data of the point. A total of 140 spectral data were collected from T1, T2, T3, and T4 treatments finally.

After the spectral acquisition, soil samples of the 0-20 cm, 20-40 cm, and 40-60 cm soil layer were collected from the spectral sampling points. In the lab, the soil samples were air-dried, ground, and sieved through a 2 mm sieve. The soil electrical conductivity (EC_1:5_) was measured with a conductivity meter (S230-K-CN, Mettler-Toledo, Switzerland) (soil: water = 1: 5). The average EC_1:5_ was calculated for the 0-20, 0-40, and 0-60 cm soil layers, which were labeled as S1, S2, and S3, respectively. Three cotton plants were collected at each spectral sampling point, and 21 cotton plants were collected for each treatment. Plant height (H) (cm) was measured in-situ with a tape measure, and cotton stems, leaves, and reproductive organs were weighted by an electronic balance. After that, the plant samples were dried at 105 °C for 30 minutes, and then dried at 80 °C to constant weight, followed by the measurement of the dry weight. The average value of three replicates was calculated. Finally, the aboveground biomass (AGB) and shoot water content (SWC) were determined.

### 2.4 Spectral preprocessing

The spectral data of 350-399 nm and 2401-2500 nm were removed because of the instrumental noises. Besides, the spectral data of 1361-1489 nm and 1811-1959 nm were also removed because these were prone to be affected by air moisture. The Savitzky–Golay Filter method was used to smooth the raw reflectance, with a window size of 13 × 13 nm. To reduce the errors generated in the spectral acquisition, the first derivative reflectance (FDR) preprocessing of the raw spectra was performed, which could reduce the influence of noise on the hyperspectral curve and the spectral difference caused by the inhomogeneity of the samples.

### 2.5 Spectral feature selection

Spectral feature selection can reduce the impact of invalid information and improve prediction accuracy and robustness and operational efficiency (Kamruzzaman et al., 2022). In this study, three methods including VIP, CARS, and RFA were used for spectral feature selection using the libPLS in Matlab (The Math Works, Inc., Natick, MA, USA).

#### 2.5.1 Variable importance in projection

VIP is a method based on the PLSR model, which reflects the explanatory capacity of independent variables on dependent variables (Oussama et al., 2012; de Almeida et al., 2013). If the VIP value of independent variable is less than 1, it indicates that the variable has little contribution to the model and has a low explanatory capacity for the dependent variable, so it can be removed in the modeling (Chavana-Bryant et al., 2019). The VIP was calculated as follows (Wold et al., 1983):


(1) $$\begin{array}{c} VIP=\sqrt{K\times \frac{\sum_{a=1}^{A}R_{d}\left(Y,t\right)W^{2}}{\sum_{a=1}^{A}R_{d}\left(Y,t\right)}} \end{array}$$

where K represents the total number of independent variables; A is the number of components; t represents the selected independent variables; R_d_ (*Y*, *t*) represents the explanatory capacity of components to dependent variables; W^2^ represents the importance of variable in each component; If the VIP value is greater than 1, it indicates a strong correlation between independent variable and dependent variable.

#### 2.5.2 Competitive adaptive reweighted sampling

CARS is a feature selection method based on the Monte Carlo sampling and PLSR coefficients. By randomly selecting part of total samples as the calibration set and establishing a PLSR model, the absolute value of the regression coefficient of the model and the corresponding weight of each band were calculated. After that, variables were selected by using the exponential decay function and the adaptive reweighted sampling method, and the root mean square error of cross-validation (RMSE_CV_) was calculated. After N times of sampling, the subset with the smallest RMSE_CV_ was selected as the optimal variable subset (Li et al., 2009; Wu et al., 2012).

#### 2.5.3 Random frog algorithm

The RFA was a variable selection algorithm based on the reversible jump Markov chain Monte Carlo methods. The selection probability of each variable was calculated by simulating a Markov Chain Monte Carlo chain that obeys the steady-state distribution in the model space. The selected wavelengths by the RFA at each run were slightly different due to Monte Carlo participation (Zhang et al., 2021b). Therefore, in order to improve the stability of the method, this study iteratively ran 100 times to determine the optimal wavelength subset by the lowest value of RMSE_CV_.

### 2.6 Modeling and evaluation

#### 2.6.1 Modelling method

The PLSR has the advantages of principal component analysis, canonical correlation analysis, and multiple linear regression analysis. This method can well deal with the problems of multicollinearity between independent variables, less sample number than variable number, and complex computation (Yang et al., 2012; Hu, 2013). In this study, the data of twenty points were randomly selected as the modeling set and the left data were set as the validation set in each stage. Therefore, 100 points were randomly selected as the modeling set and the other 40 were included in the validation set for the whole growth period. The spectral features selected by VIP, CARS, and RFA were used to extract the salinity spectral features (Spec). On this basis, cotton growth parameters were added for PLSR modeling one by one as covariates, to construct a calibration model using the Matlab software (The Math Works, Inc., Natick, MA, USA).

#### 2.6.2 Evaluation of the models

The constructed models were evaluated with coefficient of determination (R^2^), root mean square error (RMSE), and Lin’s concordance correlation coefficient (LCCC). R^2^_C_ and R^2^_V_ represent the coefficient of determination for calibration set and validation set, respectively, and RMSE_C_ and RMSE_V_ represent the root mean square error for calibration set and validation set, respectively. A satisfactory prediction generally has high R^2^_V_ and LCCC and low RMSE_V_. Besides, the closer RMSEv is to 0, the higher the prediction accuracy.


(2) $$\begin{array}{c} R^{2}=1-\frac{\sum_{i=1}^{n}{\left(y_{i}-{\hat{y}}_{i}\right)}^{2}}{\sum_{i=1}^{n}{\left(y_{i}-{\bar{y}}_{i}\right)}^{2}} \end{array}$$

(3) $$\begin{array}{c} RMSE=\sqrt{\frac{1}{n}\sum_{i=1}^{n}{\left(y_{i}-{\hat{y}}_{i}\right)}^{2}} \end{array}$$

(4) $$\begin{array}{c} LCCC=\frac{2\rho \sigma_{y}\sigma_{\hat{y}}}{\sigma_{y}^{2}+\sigma_{\hat{y}}^{2}+{\left(\mu_{y}+\mu_{\hat{y}}\right)}^{2}} \end{array}$$

Whereas *y_i_* is the predicted value of the *i*-th sample; *n* is the number of samples; *y_i_* is the average of the measured values; *ŷ_i_* is the predicted value of the *i*-th sample; *ρ* is the correlation coefficient (Pearson’s r) between measured and predicted value; *σ_y_* and *σ_ŷ_* are the variances of measured and predicted value, respectively; *μ_y_* and *μ_ŷ_* are the mean of measured and predicted value, respectively.

## 3 Results

### 3.1 Changes in cotton growth parameters and soil salinity under different saline soil amelioration treatments

Soil salinity first decreased and then increased during the whole growth period for T1, T2. T3, and T4 treatment, reaching the lowest value on 124 DAS. The average EC_1:5_ of the 0-20 cm, 0-40 cm, and 0-60 cm soil layers for different amelioration treatments on 124 DAS decreased by 3.77% ~ 16.01%, 5.00% ~ 13.89%, and 5.69% ~ 11.90%, respectively compared with those on 76 DAS (**Table 4**). On 142 and 162 DAS, due to the end of irrigation, soil salts tend to migrate upward under the force from evaporation and crop transpiration, resulting in an increase in soil salinity.

**Table 4:** Changes of soil salinity in different soil layers under different treatments (dS m^-1^).

Soil layers	DAS	T1	T2	T3	T4
0-20 cm	76 d	2.44 ± 0.44b	2.39 ± 0.39b	2.82 ± 0.28b	4.56 ± 0.93a
	111 d	2.35 ± 0.13b	2.34 ± 0.26b	2.74 ± 0.44b	4.28 ± 0.54a
	124 d	2.24 ± 0.54b	2.30 ± 0.26b	2.57 ± 0.48b	3.83 ± 0.23a
	142 d	2.33 ± 0.45c	2.35 ± 0.51c	2.86 ± 0.27b	3.87 ± 0.30a
	162 d	2.42 ± 0.55b	2.41 ± 0.29b	3.13 ± 0.65ab	3.99 ± 0.66a
0-40 cm	76 d	2.65 ± 0.52b	2.35 ± 0.37b	2.80 ± 0.46b	4.32 ± 0.88a
	111 d	2.54 ± 0.19b	2.27 ± 0.25b	2.69 ± 0.51b	3.96 ± 0.71a
	124 d	2.33 ± 0.67b	2.23 ± 0.28b	2.66 ± 0.60b	3.72 ± 0.42a
	142 d	2.47 ± 0.26c	2.29 ± 0.48c	2.85 ± 0.25b	3.80 ± 0.25a
	162 d	2.55 ± 0.53b	2.33 ± 0.34b	2.98 ± 0.69b	3.86 ± 0.73a
0-60 cm	76 d	2.81 ± 0.62bc	2.24 ± 0.34c	3.15 ± 0.52b	4.37 ± 0.80a
	111 d	2.70 ± 0.25b	2.13 ± 0.42c	2.88 ± 0.37b	4.15 ± 0.57a
	124 d	2.65 ± 0.41b	2.03 ± 0.38c	2.85 ± 0.63b	3.85 ± 0.43a
	142 d	2.79 ± 0.32c	2.21 ± 0.35d	3.24 ± 0.24b	4.25 ± 0.32a
	162 d	2.84 ± 0.52bc	2.28 ± 0.51c	3.35 ± 0.82b	4.37 ± 0.93a

The data are the average of 7 replicates; Different lowercase letters indicate significant difference between the treatments (Duncan’s test) (p< 0.05).

The four amelioration treatments reduced root zone soil salinity. The desalination effect of T2 treatment was the best, followed by T1 treatment, and the soil salinity of T3 treatment was significantly lower than that of T4 treatment. Besides, soil salinity in T1 treatment showed 0-60 > 0-40 > 0-20 cm soil layer. The soil salinity in T2 treatment showed 0-20 > 0-40 > 0-60 cm soil layer. The soil salinity in T3 and T4 treatments showed 0-60 > 0-20 > 0-40 cm soil layer.

**Figure 2:**
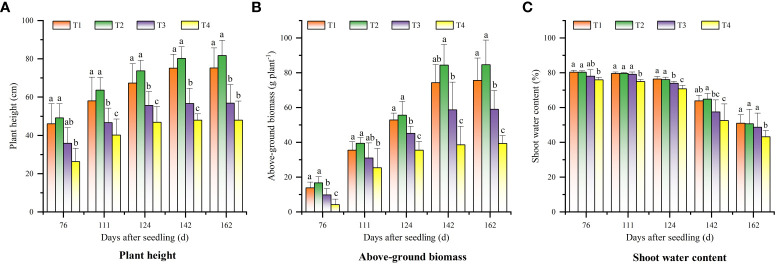
Changes of cotton growth parameters in different periods under different saline soil amelioration treatments: **(A)** plant height, **(B)** above-ground biomass, and **(C)** shoot water content.

**Figure 2A** shows the changes of cotton plant height in different sampling periods. It can be seen that the cotton plant height in the four treatments increased rapidly from 76 d to 141 DAS, peaked at 142 DAS, and stabilized from 143 to 162 DAS. The cotton plant height in each stage showed a trend of T2 > T1 > T3 > T4. Besides, there was no difference between T1 and T2 treatments (*p* > 0.05).

The aboveground biomass of cotton increased rapidly from 76 DAS (**Figure 2B**). The peaking time of aboveground biomass were not consistent in different treatments. The aboveground biomass in T1 and T2 treatments peaked at 142 DAS, while that in T3 and T4 treatments peaked at 124 DAS. The aboveground biomass of T1 and T2 treatments were higher than that of T3 and T4 treatments (*p*< 0.05), and there was no difference between T1 and T2 treatments (*p* > 0.05). The shoot water content in the treatments showed a gradual downward trend (**Figure 2C**). The shoot water content in T4 treatment was always lower than that in T1, T2, and T3 treatments (*p*< 0.05), and there was no difference between T1, T2, and T3 treatments (*p* > 0.05).

**Figure 3:**
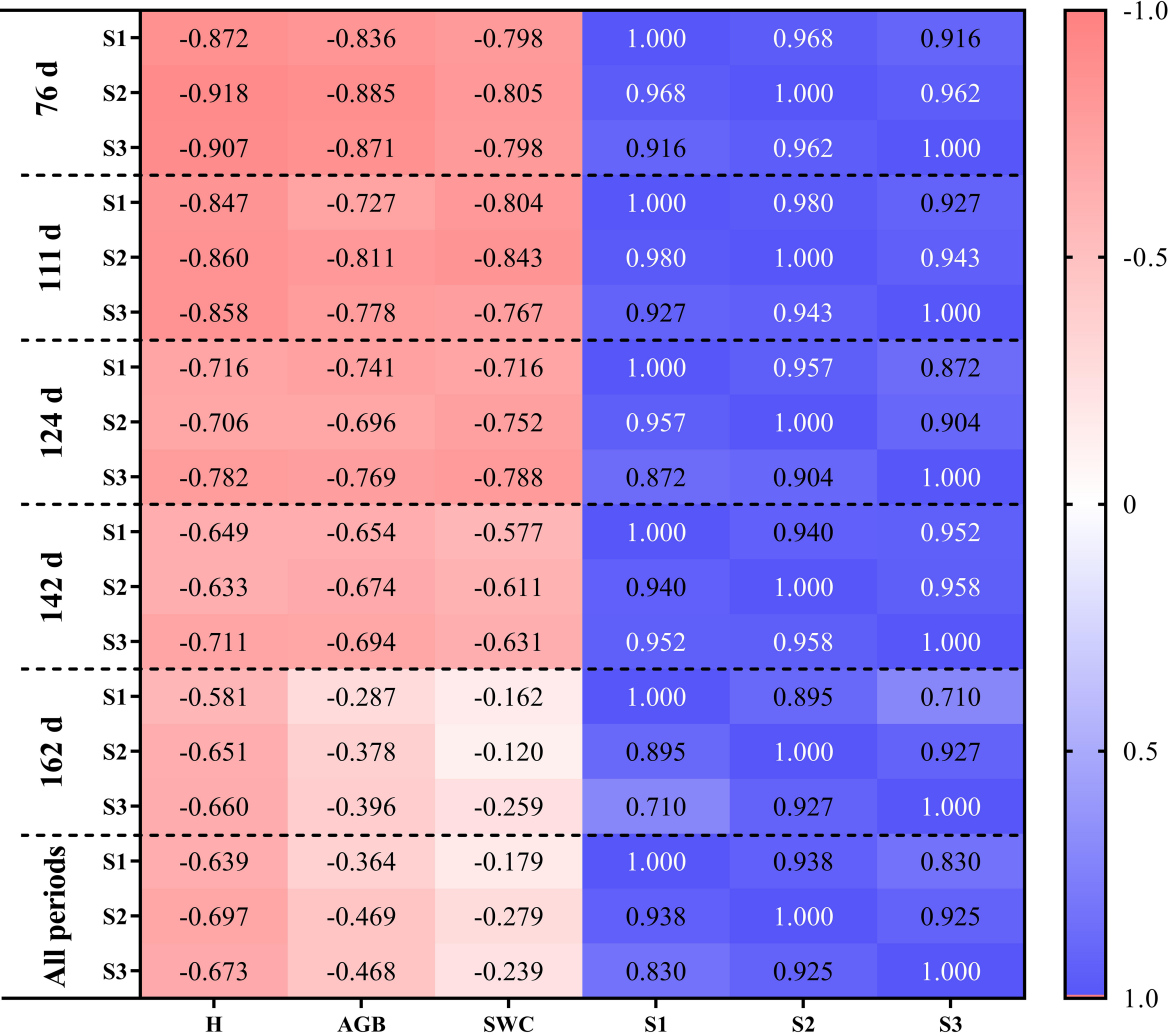
Correlation analysis between soil salinity of different soil layers and cotton growth parameters at different cotton growth periods.

There was a negative correlation between soil salinity and cotton growth parameters (cotton plant height, aboveground biomass, and shoot water content). The correlation between soil salinity and cotton growth parameters gradually weakened over time, indicating that cotton was significantly affected by soil salinity in the early stage of growth. The correlations between cotton growth parameters and S2 were the highest at 76 ~ 111 DAS, and the correlations between cotton growth parameters and S3 were the highest at 124 DAS, indicating that the salinity of 0-60 cm soil layer had an increased influence on cotton growth parameters over time. For the whole growth period, the correlation between cotton growth parameters and soil salinity was lower than that of single periods, and the correlation between cotton growth parameters and S2 was the highest. The correlation between plant height and S2 was significantly higher than that between aboveground biomass/shoot water content and soil salinity (**Figure 3**).

### 3.2 Hyperspectral changes of cotton canopy under different saline soil amelioration treatments

**Figure 4:**
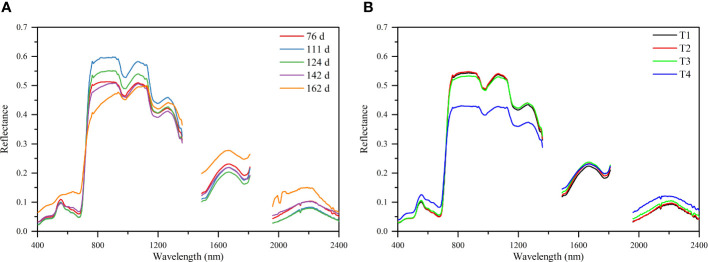
Cotton canopy hyperspectral reflectance characteristics for different periods **(A)** and different treatments **(B)**.

The canopy structure and biochemical components of cotton changed at different growth stages, leading to differences in the canopy spectra collected at different growth stages. It can be seen from **Figure 4A** that the reflectance in the Vis (visible region, 400-780 nm) and SWIR (short-wave infrared region, 780-1100 nm) showed a decreasing trend from 76 to 124 DAS, while those showed an increasing trend as the bolls opened and the blades fell off after 142 DAS. In the LWIR (Long-wave infrared, 1100-2400 nm), the reflectance increased first, peaked at 111 DAS, and then decreased. This is because cotton is mainly in the vegetation growth stage before 111 DAS, and the increase of canopy density leads to the increase of LWIR spectral reflectance. After 111 DAS, cotton enters the reproductive growth stage, and the reflectance in the LWIR decreased as leaves aged, yellowed, and dried. In addition, under different saline soil amelioration treatments, the spectral reflectance of cotton canopy were different (**Figure 4B**). With the increase of soil salinity (T2< T1< T3< T4), the spectral reflectance in the Vis increased, while that in the LWIR decreased. The difference in canopy spectral reflectance among T1, T2, and T3 treatments was small, indicating that there was little difference in canopy spectra when the soil salinity was low.

**Figure 5:**
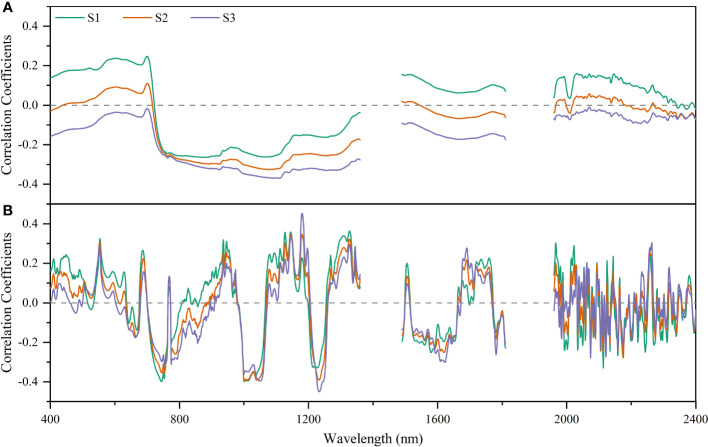
Correlation analysis of raw spectral reflectance **(A)** and first derivative reflectance **(B)** with soil salinity for different soil layers.

The correlation coefficient curves of raw spectral reflectance (R) and first derivative spectral reflectance with soil salinity in each soil layer (**Figure 5**) showed that the R had the highest correlation with S1 (r = -0.264, *p*< 0.01), S2 (r = -0.326, *p*< 0.01), and S3 (r = -0.370, *p*< 0.01) at 878, 1078, and 1112 nm, respectively (**Figure 5A**). After FDR preprocessing, the correlation coefficient curve for each soil layer changed greatly. The bands with the highest correlation with S1, S2, and S3 were at 745, 1000, and 1180 nm, with r of -0.399, -0.397, and -0.454, respectively (*p*< 0.01) (**Figure 5B**). In addition, compared with R, the absolute values of the maximum correlation coefficient between FDR and S1/S2/S3 increased by 51.14%, 21.78%, and 22.70%, respectively. That is, FDR preprocessing improved the correlation between spectral reflectance and soil salinity for each soil layer compared with R.

### 3.3 Prediction of soil salinity based on salinity spectral features

#### 3.3.1 Selection of salinity spectral features from cotton canopy spectrum

**Figure 6:**
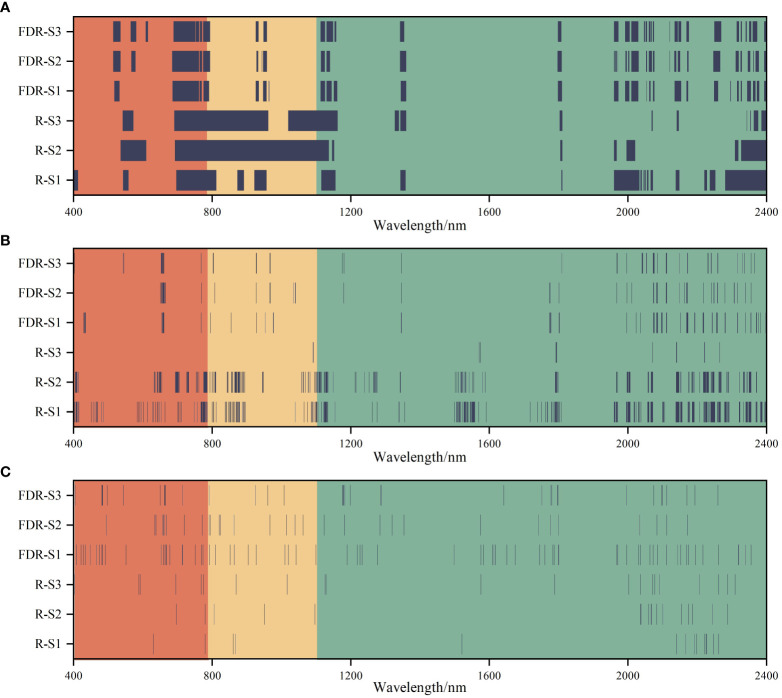
Selection of the spectral features of soil salinity based on the variable importance in projection (VIP) **(A)**, competitive adaptive reweighted sampling (CARS) **(B)**, and random frog algorithm (RFA) **(C)** feature selection methods.

The number of variables for different soil layers decreased significantly after spectral feature selection. After performing VIP, CARS, and RFA, the number of spectral features of S1, S2 and S3 were 331 ~ 649, 14 ~ 318, and 26 ~ 71, respectively (**Figure 6**). In addition, the number of spectral features selected by different methods were significantly different based on the R and FDR. For VIP and CARS, except for S3, the number of spectral features selected based on the FDR decreased by 34.97% ~ 78.37% compared with that based on the R, and the spectral features selected by RFA after FDR preprocessing were more than that based on R, especially in the range of 1100 ~ 2000 nm. Overall, CARS and RFA significantly reduced the selected variable number over a continuous range, redundancy, and collinearity.

#### 3.3.2 Soil salinity estimation based on salinity spectral features

Based on different feature selection methods, R and FDR, the PLSR models for predicting soil salinity in different soil layers were constructed (**Table 5**). The models had the highest prediction accuracy for S1, followed by S2 and S3. However, the optimal spectral preprocessing method and the optimal spectral feature selection method for soil salinity prediction were different for different soil layers. The prediction accuracy based on R for S1 and S2 were higher than that for S3, while that based on FDR for S3 was higher than that for S1 and S2. The prediction accuracy for S1 based on R + VIP was higher than that based on FDR + VIP, and prediction accuracy for S2 and S3 based on FDR + VIP was higher than that based on R + VIP. The prediction accuracy based on the CARS + FDR and RFA + FDR were higher than that based on CARS + R and RFA + R for each layer, with R^2^_v_ increasing by 6.67% - 37.50%. In general, the estimation model based on spectral features had higher accuracy than that based on full spectra, among which the CARS method was the optimal spectral feature selection method for the R and FDR (R^2^_v_: 0.416 ~ 0.659), followed by RFA. The prediction accuracy of the estimation model based on the spectral features selected by VIP was only slightly higher than that based on the full spectra. Therefore, in the subsequent analysis, the spectral features selected by CARS were used for soil salinity estimation.

**Table 5:** Model accuracy evaluation of soil salinity based on canopy hyperspectral data.

Index screening method	Soil layer/cm	R						FDR					
		LV	R^2^_C_	RMSE_C_	R^2^_V_	RMSE_V_	LCCC	LV	R^2^_C_	RMSE_C_	R^2^_V_	RMSE_V_	LCCC
Full spectra	0-20	9	0.517	0.637	0.413	0.658	0.679	4	0.473	0.665	0.334	0.701	0.605
	0-40	7	0.474	0.607	0.313	0.631	0.641	3	0.391	0.653	0.293	0.640	0.519
	0-60	4	0.331	0.691	0.242	0.680	0.517	4	0.413	0.648	0.289	0.658	0.492
VIP	0-20	4	0.528	0.630	0.416	0.656	0.696	5	0.462	0.672	0.373	0.681	0.620
	0-40	5	0.376	0.661	0.348	0.614	0.627	3	0.448	0.622	0.359	0.609	0.597
	0-60	9	0.515	0.588	0.248	0.677	0.581	4	0.488	0.605	0.310	0.649	0.556
CARS	0-20	9	0.593	0.585	0.516	0.598	0.737	6	0.735	0.472	0.659	0.456	0.802
	0-40	8	0.571	0.548	0.499	0.539	0.736	5	0.704	0.455	0.626	0.526	0.813
	0-60	9	0.428	0.639	0.416	0.597	0.621	5	0.667	0.488	0.572	0.498	0.77
RFA	0-20	10	0.496	0.651	0.495	0.611	0.736	5	0.581	0.593	0.528	0.590	0.762
	0-40	10	0.541	0.567	0.429	0.575	0.675	7	0.579	0.543	0.483	0.547	0.735
	0-60	4	0.422	0.643	0.383	0.613	0.623	4	0.570	0.554	0.499	0.553	0.720

### 3.4 Soil salinity estimation based on the combination of canopy spectral features and plant growth parameters

**Figure 7:**
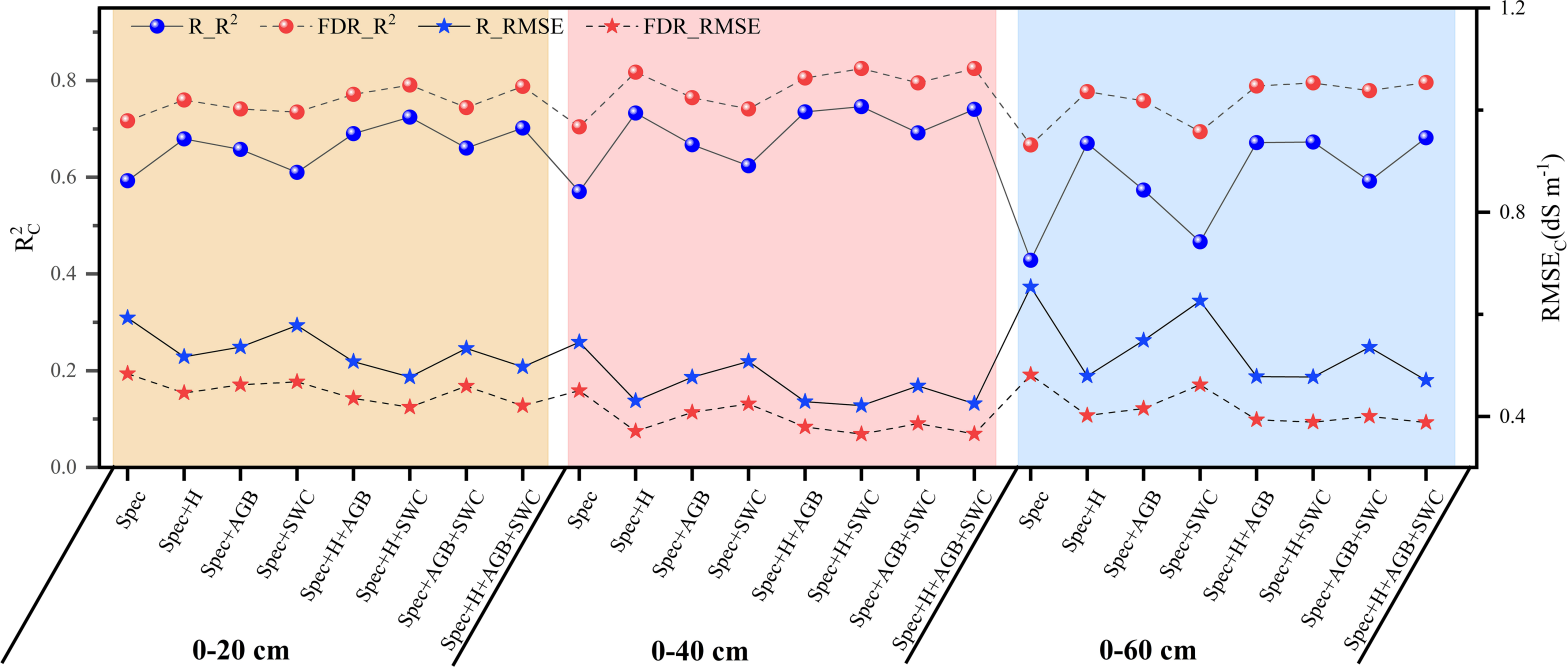
Soil salinity prediction accuracy of the model based on the combination of spectral features selected by competitive adaptive reweighted sampling and plant growth parameters.

The average soil salinity estimation accuracy for the three soil layers based on the spectral features selected based on the FDR increased by 20.88%, 23.44%, and 55.71%, respectively compared with that based on the spectral features selected based on the R (**Figure 7**). FDR was better than R in the prediction of soil salinity for each soil layer. When plant growth parameters were included in predictor variables for salinity prediction, the prediction accuracy of the model (R^2^_C_: 0.466-0.825) was higher than that of the model constructed using spectral features only (R^2^_C_: 0.428-0.717). Therefore, the combination of plant growth parameters and soil salinity spectral features could effectively improve the prediction accuracy. For the 0-20 cm, 0-40 cm, and 0-60 cm soil layer, the R^2^_C_ of the model constructed based on the combination of spectral features and plant growth parameters increased by 2.53% ~ 10.34%, 5.27% ~ 17.15%, and 4.09% ~ 19.37%, respectively compared with that of the model constructed using spectral features only. In addition, among all the prediction models, the models constructed based on Spec_FDR_+H+SWC and Spec_FDR_+H+AGB+SWC after the FDR preprocessing performed equally well for predicting soil salinity in each layer, so the two models were further validated independently.

**Figure 8:**
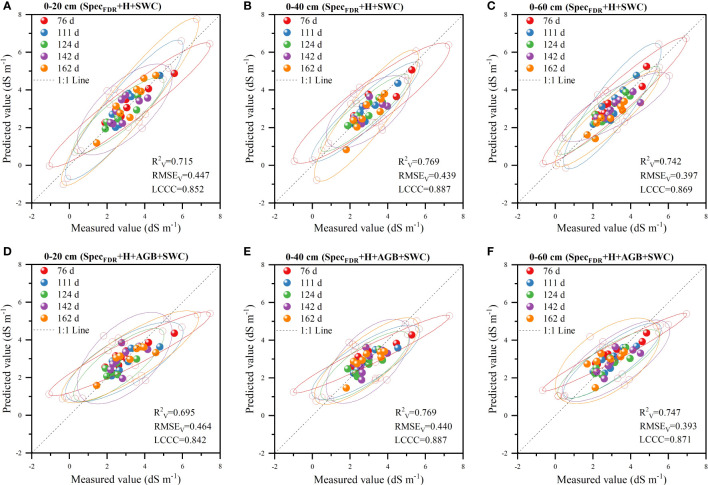
Validation of prediction accuracy of the Spec^FDR^+H+SWC model (**A**–**C** represents 0-20 cm, 0-40 cm and 0-60 cm respectively) and Spec^FDR^+H+AGB+SWC model (**D**–**F** represents 0-20 cm, 0-40 cm and 0-60 cm respectively) (n = 40; Ellipses denote the 95% confidence intervals for each period; The long-dashed black line is the 1:1 line).

The validation results of the two models (Spec_FDR_+H+SWC and Spec_FDR_+H+AGB+SWC) (**Figure 8**) showed that the models had the highest prediction accuracy for S2, followed by S3 and S1. Meanwhile, the comparison of the prediction accuracy of the two models for each soil layer showed that for S3, the prediction accuracy of the model based on Spec_FDR_+H+AGB+SWC was higher than that of the model based on Spec_FDR_+H+SWC data (R^2^_V_ and LCCC increased by 0.005 and 0.002 respectively, and RMSE_V_ decreased by 0.004 dS m^-1^). There was no difference in the accuracy between the two models for S2. Besides, for S1, the prediction accuracy of the Spec_FDR_+H+AGB+SWC model was slightly lower than that of the Spec_FDR_+H+SWC model. Therefore, the addition of AGB did no increase the prediction accuracy of soil salinity in each layer, but it reduced the prediction accuracy of soil salinity in the 0-20 cm layer. Therefore, the optimal soil salinity prediction model was the Spec_FDR_+H+SWC model.

## 4 Discussion

### 4.1 Effects of soil salinity on growth parameters of cotton

Under high soil salinity conditions, crop growth and development are greatly inhibited (Kibria and Hoque, 2019; Litalien and Zeeb, 2020), which is directly reflected in plant height, dry matter yield, moisture content, etc. Guo et al. (2019) reported that the growth of roots, stems, and leaves of cotton was increasingly inhibited with increasing NaCl concentration. Moussa et al. (2019) reported that salt stress led to a decrease in plant height, leaf number, leaf area, specific leaf area, and root dry matter accumulation of wheat. Soil salinity first decreased and then increased during the whole growth period in this study, which reached the lowest on 124 DAS. This may be due to that high frequency of irrigation during cotton growth period is conducive to reducing soil salinity in the plough layer, and film mulching can inhibit the upward movement of the salts in the deep soil layer by reducing the surface evaporation. The difference in soil salinity under different treatments caused significant differences in cotton growth parameters (H, AGB, and PWC) (**Figure 3**). Generally, cotton plant height, aboveground biomass, and aboveground moisture content decreased with the increase of soil salinity. This is consistent with the findings of Ahmad et al. (2002). Studies have shown that the growth parameters of crops, such as aboveground fresh weight, plant height, shoot water content, and physiological related indicators, are sensitive to soil salinity (AbdelRahman et al., 2019). In this study, cotton plant height was most affected by soil salinity. This may be due to that on the one hand, high soil salinity could reduce soil water potential, resulting in reduced plant water potential and increased water loss (Nawaz et al., 2013). On the other hand, excessive salt ions in soil can cause leaf cell damage during transpiration, resulting in a decrease in plant photosynthesis rate, abnormal plant growth, and low plant height (Greenway and Munns, 1980). In addition, cotton has different tolerance to salinity stress at different growth stages. Ahmad et al. (2002) and Wang et al. (2011) found that cotton was more tolerant to salt stress at the germination stage than at the seedling stage. This study found that the correlation between soil salinity and cotton growth parameters gradually weakened over time. This indicates that the inhibition effect of soil salinity on cotton growth was more obvious in the early growth stage of cotton. With the accumulation of crop photosynthetic product, cotton’s tolerance to salt stress increases (Greenway and Munns, 1980).

### 4.2 Effect of spectral feature selection methods on soil salinity prediction accuracy

Spectral data contains massive information, and how to extract useful information from massive and high-dimensional data is a big challenge for current spectral data analysis (Peng et al., 2020). Spectral feature selection can remove redundancies in the spectrum and improve the prediction capacity. In this study, the effects of VIP, CARS, and RFA methods on the soil salinity prediction accuracy of three soil layers showed that the prediction accuracy of the model based on spectral features was higher than that of the model based on the full band. Compared with the VIP, the CARS and RFA selected fewer spectral features (**Figure 6**). This may be due to the difference in the search mechanism of the three methods (Lao et al., 2020; Sun et al., 2021b). CARS could select the bands with large absolute value of regression coefficient in PLSR model, and the variables with smaller weight were removed. This method could select the optimal band set closely related to soil salinity. RFA selects spectral features by the frequency of being selected. VIP judges the explanatory capacity of independent variables through the principal components of independent variables (Xing et al., 2019), and retains as many variables as possible. Besides, it was found that the CARS and RFA were superior to VIP in predicting soil salinity of different soil layers. The less and representative selected variables could reduce the complexity of modeling and make the prediction model more stable. This is consistent with the results of many previous studies. For example, Lao et al. (2020) used VIP, CARS and RFA methods to select soil salinity spectral features, and found that compared with the full spectrum (root mean square error of prediction RMSEp = 2.54%) and the variables screened by VIP (RMSEp = 2.17%), the RFA method had the most obvious improvement in the accuracy of in-situ spectral estimation of soil salinity (RMSEp = 1.63%, residual prediction deviation RPD = 3.80), followed by the CARS method (RMSEp = 2.0%, RPD = 3.09). Sun et al. (2021b) used VIP, RFA, and CARS to select spectral features to predict maize leaf water content, and found that compared with the PLS model constructed based on the VIP method, the PLS model constructed based on the CARS and RFA methods had higher prediction accuracy, because CARS and RFA methods could reduce the complexity of the model, extract more important information related to maize leaf water content.

Spectral feature extraction can reduce the number of spectral variables and improve prediction accuracy (Zou et al., 2010; Yun et al., 2019), but there are still some problems. This research proved that proper data preprocessing can enhance spectral quality and facilitate the extraction of spectral information. However, it is unclear how spectral data preprocessing affects the extraction of key spectral variables (Gholizadeh et al., 2021). At present, the consensus on the spectral feature extraction algorithm is that the extraction of key variables streamlining the modeling variables by eliminating redundant variables. But the stability and reliability of the selected variables and the universality of the model still one of the contents that need to be further studied.

### 4.3 Soil salinity prediction accuracy of the model based on the combination of spectral features and plant growth parameters

**Figure 9:**
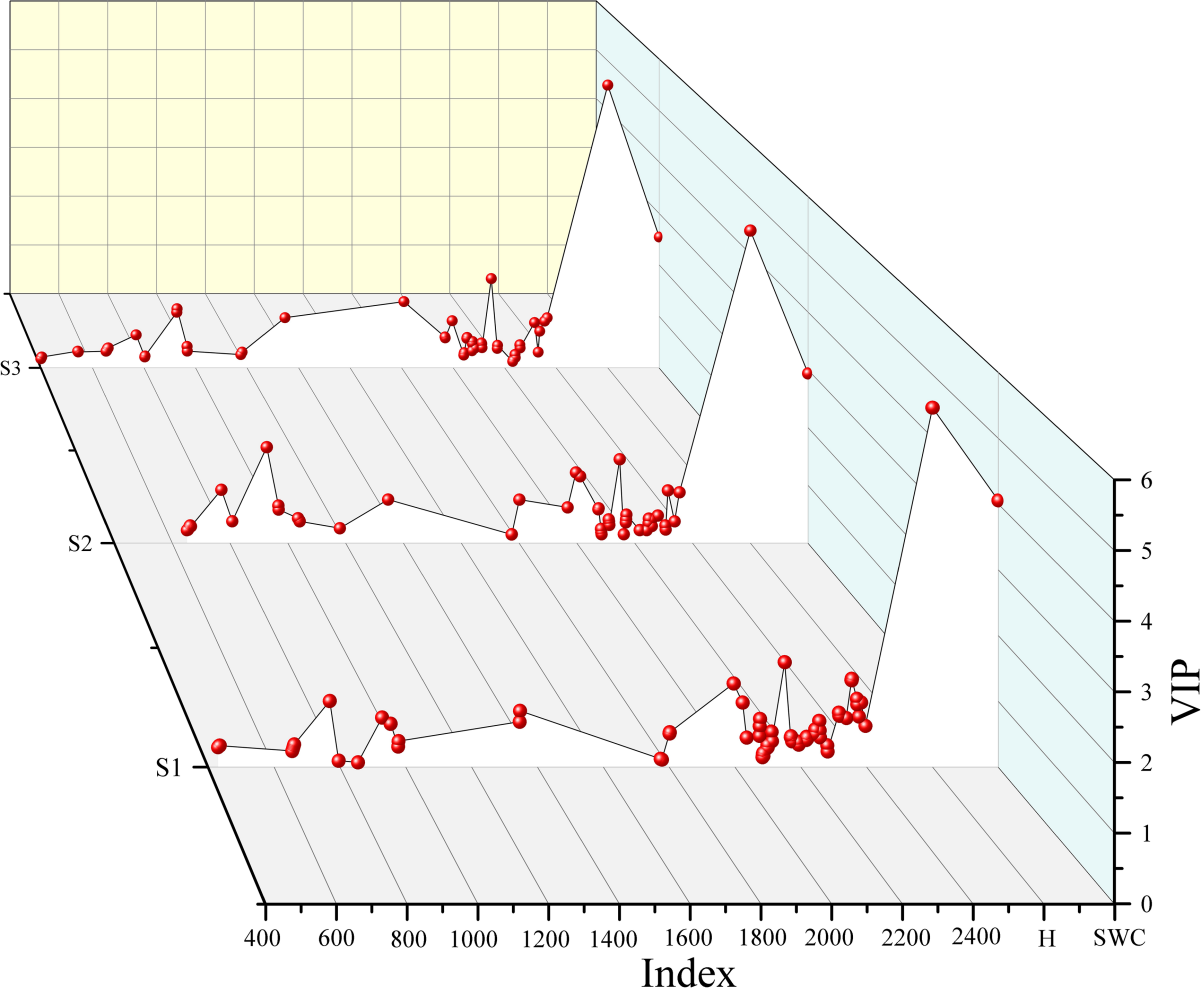
Analysis of the importance of all variables in the Spec_FDR_+H+SWC model.

The interference of vegetation cover on soil spectrum is one of the main limiting factors in the estimation of soil attributes by remote sensing technology. Therefore, exploring the method for eliminating vegetation cover area or enhancing soil spectral characterization by collaborating with vegetation spectral information is of great significance for improving the accuracy of soil attribute inversion by remote sensing (Zhang et al., 2021a). Differences in soil salinity can lead to differences in landscape (vegetation), and many studies have used hyperspectral reflectance of vegetation canopy or derived vegetation indices such as normalized difference vegetation index (NDVI) (Garajeh et al., 2021; Gómez Flores et al., 2022), photochemical reflectance index (PRI) (Ivushkin et al., 2019; Kim et al., 2020) etc., to indirectly estimate soil salinity. However, the vegetation canopy reflectance spectrum is mixed with the spectrum of ground objects, and affected by factors such as leaf characteristics, canopy structure, soil properties, and atmospheric conditions (Guo et al., 2017). Especially, the vegetation index has little variation when the vegetation grows densely, which limits the prediction accuracy of soil salinity based on canopy spectral reflectance and vegetation index (Daughtry et al., 2000; Peng et al., 2020; Ramos et al., 2020). The results of this study showed that the prediction accuracy of the model based on the combination of plant growth parameters and spectral features was higher than that of the model based on only the spectral features. Therefore, the auxiliary variables could improve the soil salinity prediction accuracy. The relative importance of the variables for soil salinity estimation for different soil layers (**Figure 9**) showed that cotton plant height was the main factor to improve the estimation accuracy. Besides, cotton shoot water content is also closely related to soil salinity.

This study used consider plant height, dry matter yield, and plant water content as the variables for soil salinity prediction. However, changes in soil salinity can also lead to changes in other plant parameters such as chlorophyll content, photosynthesis, and canopy structure. Therefore, the contribution of different plant growth parameters to soil attribute prediction accuracy will be compared in our future study.

## 5 Conclusion

Hyperspectral technology has been widely used in the monitoring of soil salinity. However, the interference of vegetation cover greatly limits the hyperspectral monitoring of root zone soil salinity. This study proposed a new method for estimating soil salinity of different soil layers under vegetation cover conditions.

The canopy spectra were acquired to extract spectral features by VIP, CARS, and RFA methods after spectral preprocessing, and then the spectral features were combined with crop growth parameters (cotton plant height, aboveground dry matter, and shoot water content) to construct PLSR model for predicting soil salinity of different soil layers. The results showed that the soil salinity of the 0-20, 20-40, and 40-60 cm soil layers in the cotton field showed a trend of decreased first and then increased during the whole growth period, and soil salinity was negatively correlated with cotton plant height, aboveground biomass, and shoot water content. The correlation between soil salinity and cotton growth parameters gradually weakened over time. The CARS method was the most effective method, which not only reduced the proportion of selected bands to 18.46% of the full spectra, but also improved the prediction accuracy. Besides, the combination of spectral features and cotton growth parameters significantly improved the prediction accuracy of soil salinity of the 0-20, 0-40, and 0-60 cm soil layer, compared with the prediction based on the spectral features only, with R^2^ increased by 10.01%, 18.35%, and 29.90%, respectively. This method has a great application potential in the monitoring of soil salinity in the root zone during crop growth period.

The combination of cotton canopy spectrum and plant growth parameters can improve the spectral estimation accuracy of soil salinity in the root zone in cotton fields. However, near-ground hyperspectral technology still has limitations in farmland-scale information collection due to the limitation of data acquisition methods. Compared with ground based hyperspectral imaging, UAV-based remote sensing has higher spatial resolution, and is more convenient and flexible, which provides the possibility for large-scale and continuous acquisition of crop growth information. Therefore, we will try to verify the potential of combining UAV-based hyperspectral images with plant growth parameters in soil salinity monitoring, to realize rapid real-time and accurate monitoring of soil salinity at the farmland scale. This study provides new ideas for spectral estimation of soil attributes based on UAV remote sensing.

## Data availability statement

The data analyzed in this study is subject to the following licenses/restrictions: The raw data supporting the conclusions of this article are available on request to the corresponding authors.

## Author contributions

Conceptualization: XS, JS and HW. Methodology: XS and JS. Investigation: XS, JS, TT, JW, WL, MZ and MJ. Writing—original draft preparation: XS and JS. Writing—review and editing: XS, HW and XL. Funding acquisition: HW. All authors have read and agreed to the published version of the manuscript. All authors contributed to the article and approved the submitted version.
